# Multidimensional Analysis of PANoptosis-Related Molecule CASP8: Prognostic Significance, Immune Microenvironment Effect, and Therapeutic Implications in Hepatocellular Carcinoma

**DOI:** 10.1155/2023/2406193

**Published:** 2023-12-30

**Authors:** Fei Peng, Fang Zhu, Baodi Cao, Liang Peng

**Affiliations:** The Second People's Hospital of Jingdezhen, Jingdezhen 333000, Jiangxi, China

## Abstract

**Background:**

Hepatocellular carcinoma (HCC) presents significant challenges in diagnosis and treatment. Understanding the role of PANoptosis-related molecules in HCC is crucial for advancing therapeutic strategies.

**Methods:**

We conducted a comprehensive analysis using public data from the Cancer Genome Atlas, Human Protein Atlas, Tumor Immune Single Cell Hub, and STRING databases. Techniques included Kaplan–Meier survival curves, Cox regression, LASSO analysis, and various computational methods for understanding the tumor microenvironment. We also employed ClueGO, gene set enrichment analysis, and other algorithms for biological enrichment analysis.

**Results:**

CASP8 emerged as a significant molecule in HCC, correlated with poor survival outcomes. Its expression was predominant in the nucleoplasm and cytosol and varied across different cancer types. Biological enrichment analysis revealed CASP8's association with critical cellular activities and immune responses. In the tumor microenvironment, CASP8 showed correlations with various immune cell types. A nomogram plot was developed for better clinical prognostication. Mutation analysis indicated a higher frequency of TP53 mutations in patients with elevated CASP8 expression. In addition, CASP8 was found to regulate YEATS2 in HCC, highlighting a potential pathway in tumor progression.

**Conclusions:**

Our study underscores the multifaceted role of CASP8 in HCC, emphasizing its prognostic and therapeutic significance. The regulatory relationship between CASP8 and YEATS2 opens new avenues for understanding HCC pathogenesis and treatment strategies.

## 1. Introduction

Liver cancer, particularly hepatocellular carcinoma (HCC), represents one of the most lethal malignancies worldwide, posing a significant public health challenge [1]. The incidence and mortality rates of this cancer are on a continuous rise globally, with a pronounced prevalence in Asia and Africa [2]. The high incidence of liver cancer is closely associated with factors such as hepatitis virus infection, alcohol consumption, obesity, and environmental contributors [3]. The prognosis for patients with liver cancer is generally poor, primarily due to the advanced stage of the disease at diagnosis and the cancer's poor response to traditional chemotherapy and radiotherapy [4]. These challenges have spurred extensive research into more effective treatment modalities for liver cancer, particularly in the realm of molecular targeted therapy [5]. Significant advancements have been made in molecular targeted therapies in recent years. This therapeutic approach is predicated on a deep understanding of the biological characteristics of tumor cells and aims to develop drugs that target specific molecular markers [6]. For instance, targeted therapies developed against common signaling pathway aberrations in liver cancer cells, such as the PI3K/AKT/mTOR and RAS/RAF/MEK/ERK pathways, have shown promising therapeutic potential [7, 8]. In addition, immunotherapy, especially the use of immune checkpoint inhibitors, has demonstrated unprecedented potential in the treatment of liver cancer [9]. However, further research is needed to determine which patients will benefit from these therapies and how to combine these treatments to enhance efficacy. Consequently, research on liver cancer is focusing on improving early diagnostic methods, understanding the molecular biological mechanisms of the tumor, and developing more effective personalized treatment strategies.

PANoptosis represents a comprehensive concept encompassing various forms of programmed cell death, including but not limited to classical apoptosis, necroptosis, ferroptosis, and pyroptosis [10]. This notion is crucial for understanding the diversity and complexity of cell death, especially in disease states. In the field of oncology, research into PANoptosis offers novel insights into the mechanisms of survival and death of tumor cells, with particular emphasis on HCC [11]. HCC, a highly aggressive tumor, often exhibits dysregulation in its cell death pathways, which are intricately linked to the tumor's development, progression, and resistance to treatment [12]. Current research in HCC focuses on exploring how tumor cells evade programmed cell death and utilizing this knowledge to develop novel therapeutic strategies. For instance, studies have shown that HCC cells evade apoptosis by altering specific apoptotic signaling pathways, such as the expression of Bcl-2 family proteins, thus affecting tumor growth and spread [13]. Targeted therapies against these pathways could offer new avenues for HCC treatment. In addition, understanding the unique mechanisms of PANoptosis in HCC is vital for predicting treatment responses and developing personalized treatment plans. Given the central role of PANoptosis in the pathogenesis of HCC, in-depth research in this area is crucial for the development of new diagnostic biomarkers and therapeutic targets [14]. This will not only help elucidate the pathophysiological mechanisms of HCC but may also lead to more effective treatment modalities, thereby improving clinical outcomes for patients.

Our study conducted a thorough investigation of CASP8 in HCC. We identified CASP8 as a crucial molecule through analyses of PANoptosis-related molecules, ClueGO, and protein-protein interaction networks. High CASP8 expression was linked to poor patient survival, suggesting its prognostic importance. Immunofluorescence images confirmed CASP8's presence in the nucleoplasm and cytosol. A pan-cancer analysis and single-cell techniques revealed its varied expression and distribution across different cancer types and cell types, respectively. Biological enrichment analysis showed CASP8's association with various biological pathways, and its correlation with diverse immune cell types indicated a significant role in the HCC immune landscape. In addition, we developed a nomogram for better clinical prognosis, analyzed CASP8's influence on immunotherapy and chemotherapy, and investigated its relationship with TP53 mutations. The study also explored CASP8's regulatory effect on YEATS2, further understanding its biological significance in HCC. This research underscores the potential of CASP8 as a key prognostic marker and therapeutic target in HCC.

## 2. Methods

### 2.1. Downloading and Preprocessing Public Data for HCC

Initially, the clinical data, expression profiles, and mutation data were acquired from The Cancer Genome Atlas (TCGA) database [15]. The format of the expression profiles was STAR-Counts, which was subsequently converted into the TPM format using the author's R code. The clinical data were formatted in bcr-xml. Prior to data analysis, the expression profile data underwent preprocessing, including normalization, to ensure data quality and reproducibility. Representative cell fluorescence images were sourced from the Human Protein Atlas (HPA) database [16]. In addition, single-cell data were downloaded from the Tumor Immune Single Cell Hub (TISCH) project [17], a comprehensive database that provides detailed single-cell expression profiles to facilitate the study of tumor immunology and the tumor microenvironment across various cancer types [18–23]. The data of protein interaction were obtained from the STRING database.

### 2.2. Prognosis Analysis

The prognosis analysis of our study focuses on evaluating the survival outcomes of patients using Kaplan–Meier (KM) survival curves and univariate Cox regression analysis. The KM survival curves are employed to graphically represent the survival probability over time, allowing us to visually compare the survival experiences of different patient cohorts. This method is instrumental in estimating survival functions and median survival times. On the other hand, univariate Cox regression analysis is used to assess the impact of individual variables on survival. To further improve the clinical potential of CASP8 in HCC, we develop a nomogram. This graphical tool integrates multiple prognostic variables, identified as significant in the Cox regression analysis, into a single model. LASSO regression analysis is a penalized regression method effective in handling high-dimensional data and in selecting the most relevant prognostic factors while controlling for overfitting.

### 2.3. Biological Enrichment Analysis

We employ both ClueGO and gene set enrichment analysis (GSEA) to elucidate the biological contexts and pathways [24]. ClueGO, a Cytoscape plugin, is utilized for deciphering functionally grouped gene ontology and pathway annotation networks [25]. In addition, GSEA is performed to identify whether a predefined set of genes shows statistically significant, concordant differences between two biological states. It helps in understanding gene expression data at the level of gene sets, based on their distribution within ranked gene lists, thereby offering a more comprehensive view of the biological pathways and processes involved.

### 2.4. Tumor Microenvironment and Immune Function Analysis

Our study integrates a suite of sophisticated computational methods to characterize the cellular composition and immune landscape within the tumor milieu. We utilize CIBERSORT, an algorithm that applies a deconvolution method to estimate the cell type proportions in bulk tissue gene expression data, providing insights into the immune cell composition [26]. EPIC is employed for quantifying the abundance of stromal and immune cells in tumor samples [27]. MCPcounter is used for the robust quantification of the presence of specific immune and stromal cell populations [28]. QUANTISEQ, an algorithm designed for immune profiling, is applied to dissect the tumor immune contexture [29]. TIMER, another critical tool in our analysis, is utilized for the systematic evaluation of tumor-infiltrating immune cells and their clinical implications [30]. xCell, a gene signature-based method, aids in the comprehensive profiling of the tumor microenvironment, encompassing a wide variety of immune and stromal cell types [31]. The immune function status was quantified using the single-sample GSEA (ssGSEA) algorithm [32].

### 2.5. Statistical Analysis

We apply a rigorous and systematic approach, ensuring the validity and reliability of our findings, with all statistical analyses and graph creation conducted using the R programming language. Initially, we assess the normality of data using the Shapiro–Wilk test. For data that follow a normal distribution, parametric tests such as the Student's *t*-test (for two-group comparisons) or one-way ANOVA (for comparing multiple groups) are used. Conversely, for nonnormally distributed data, we employ nonparametric tests such as the Mann–Whitney *U* test (for two groups) or the Kruskal–Wallis test (for more than two groups). All statistical tests are two-sided, and a significance threshold is set at a *p* value of less than 0.05.

## 3. Results

### 3.1. Identification of PANoptosis-Related Molecule CASP8 in HCC

**Figure 1:**
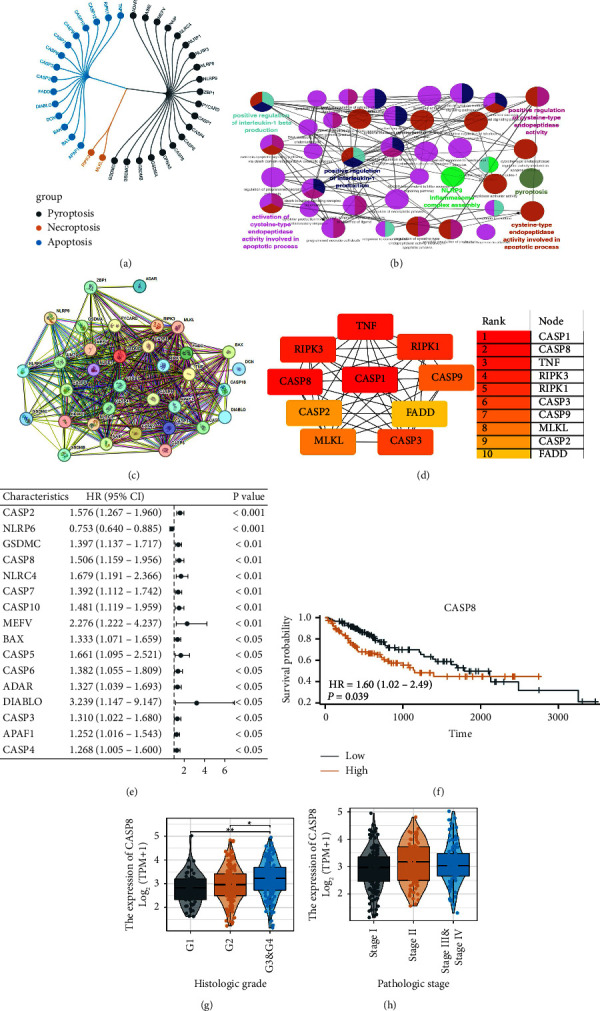
Identification of the PANoptosis-related molecule CASP8 in HCC. (a) List of the molecules of PANoptosis collected from the previous studies; (b) ClueGO analysis of these PANoptosis-related molecules; (c) PPI network of these PANoptosis-related molecules; (d) key nodes of these PANoptosis-related molecules; (e) univariate Cox regression analysis of these PANoptosis-related molecules; (f) KM survival curves of CASP8 in HCC; (g–h) the expression level of CASP8 in patients with different clinical stages.

In our study, we began by compiling a comprehensive list of molecules associated with PANoptosis, guided by extensive previous research in this field (Figure 1(a)). To further explore their biological significance, we employed ClueGO analysis, revealing that these molecules predominantly contribute to various biological processes. These include the positive regulation of interleukin-1 beta production, enhancement of cysteine-type endopeptidase activity, and the promotion of interleukin-1 production. In addition, they are involved in pyroptosis and the activation of cysteine-type endopeptidase activity in apoptotic processes (Figure 1(b)). To understand the intricate interactions among these molecules, we constructed a protein-protein interaction (PPI) network. This network highlighted the complex interplay and revealed the top ten central nodes as TNF, RIPK3, CASP8, CASP1, RIPK1, CASP9, CASP2, FADD, MLKL, and CASP3 (Figures 1(c)–1(d)). Following this, we applied univariate Cox regression analysis to discern the prognostic significance of these molecules in HCC (Figure 1(e)). This analysis identified several molecules as potential risk factors, including CASP2, GSDMC, CASP8, NLRC4, and others, while NLRP6 emerged as a protective factor. Our focused attention then shifted to CASP8, which is singled out for its prominent role in the PPI network and its statistical significance in the Cox regression analysis. We discovered that high expression levels of CASP8 in HCC patients correlated with poorer survival outcomes, as depicted in the KM survival curve (Figure 1(f)). In addition, our observations suggested a potential link between elevated CASP8 levels and worsened histological grades, although no significant correlation with clinical staging was observed (Figures 1(g)–1(h)).

### 3.2. Expression Pattern of CASP8 in HCC

**Figure 2:**
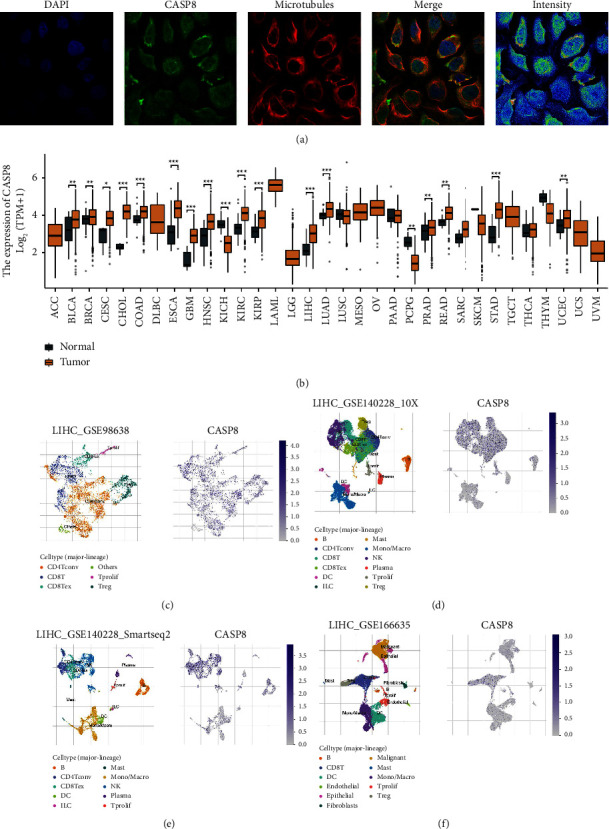
Expression level of CASP8 in HCC. (a): subcellular localization of CASP8 in the cell line obtained from the HPA database; (b) the expression level of CASP8 in pan-cancer; (c–f): the expression pattern of CASP8 in the HCC microenvironment at the single-cell level.

Utilizing representative immunofluorescence images from the HPA database, our investigation revealed the cellular localization of CASP8. The images clearly showed that CASP8 is primarily located in the nucleoplasm and cytosol, which suggests a significant role for CASP8 in these specific cellular regions (Figure 2(a)). Delving further into the expression profile of CASP8, we extended our research to a pan-cancer analysis. This comprehensive examination indicated that CASP8 exhibits varied expression levels across multiple cancer types, highlighting its potential importance in the pathogenesis of a diverse range of cancers (Figure 2(b)). To gain a more detailed understanding of CASP8 distribution, we employed single-cell analysis techniques. The results of this intricate examination revealed that CASP8 is extensively distributed across a wide array of cell types (Figures 2(c)–2(f)). This widespread presence emphasizes the versatility of CASP8 in cellular processes, potentially affecting various aspects of cancer biology.

### 3.3. Biological Enrichment Analysis

**Figure 3:**
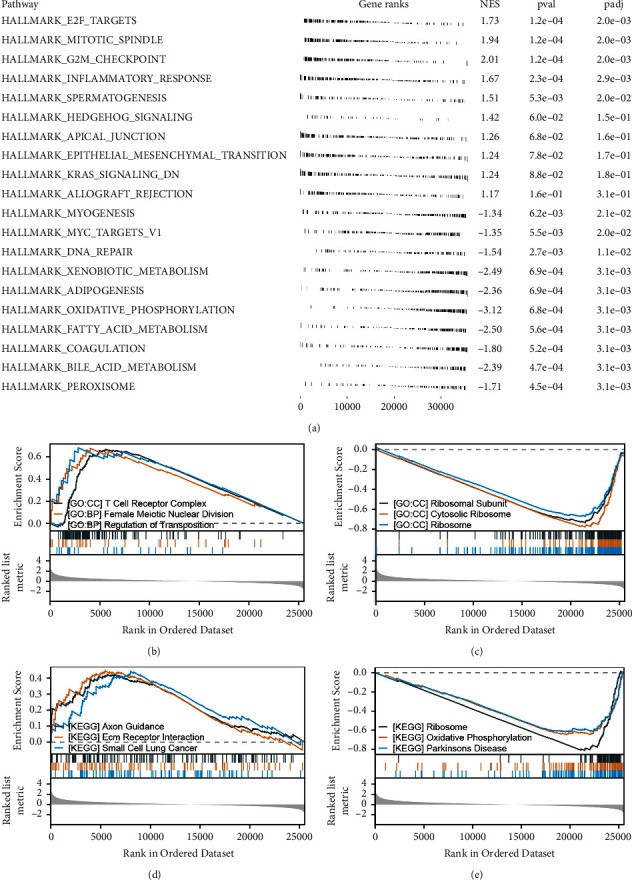
Biological enrichment of CASP8. (a) GSEA of CASP8 in HCC based on the hallmark gene set; (b) the top three upregulated GO terms of CASP8 based on GSEA; (c) the top three downregulated GO terms of CASP8 based on GSEA; (d) the top three upregulated KEGG terms of CASP8 based on GSEA; (e) the top three downregulated KEGG terms of CASP8 based on GSEA.

GSEA provided significant insights into the biological pathways and processes associated with CASP8. We found that CASP8 expression positively correlates with several key cellular activities. These include the E2F target activity, processes related to the mitotic spindle and G2M checkpoint, inflammatory responses, spermatogenesis, Hedgehog signaling pathways, apical junction mechanisms, and the epithelial-mesenchymal transition (EMT). Conversely, CASP8 showed a negative correlation with activities such as myogenesis, MYC target processes, DNA repair, xenobiotic metabolism, adipogenesis, oxidative phosphorylation, and fatty acid metabolism (Figure 3(a)). In terms of Gene Ontology (GO) terms, our GSEA revealed that patients with high CASP8 expression demonstrated increased activity in several biological processes. Notably, there was heightened activity in the T cell receptor complex, female meiotic nuclear division, and regulation of transposition. On the other hand, these patients exhibited reduced activity in areas such as ribosomal subunits, cytosolic ribosomes, and ribosomes in general (Figures 3(b)–3(c)). For Kyoto Encyclopedia of Genes and Genomes (KEGG) pathways, GSEA further highlighted that high CASP8 expression in patients was linked to increased activity in axon guidance, extracellular matrix (ECM) receptor interactions, and pathways relevant to small cell lung cancer. In contrast, there was a notable decrease in activities related to the ribosome, oxidative phosphorylation, and pathways associated with Parkinson's disease (Figures 3(d)–3(e)).

### 3.4. Immune Microenvironment Analysis

**Figure 4:**
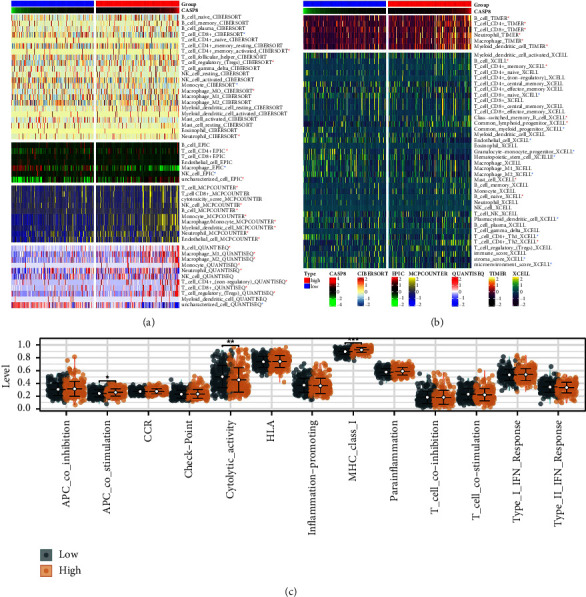
Immune analysis of CASP8. (a–b) Correlation between CASP8 and the cell components in HCC quantified by multiple algorithms and (c) difference of immune terms quantified by ssGSEA algorithms in patients with high and low CASP8 expression.

Delving into the influence of CASP8 on the tumor microenvironment of HCC, our research uncovered significant correlations with various immune cell types. CASP8 exhibited a positive relationship with memory activated CD4+ T cells, T regulatory cells (Tregs), neutrophils, populations of CD4+ and CD8+ T cells, natural killer (NK) cells, B cells, endothelial cells, mast cells, and naive B cells. In contrast, a negative correlation was observed with M2 macrophages, CD4+ Th1 T cells, and naive CD8+ T cells (Figures 4(a) and 4(b)). In addition, the analysis indicated that patients with high CASP8 expression might have elevated levels of major histocompatibility complex class I (MHC class I), juxtaposed with lower cytolytic activity (Figure 4(c)). This suggests a complex interaction between CASP8 expression and the immune response within the TME of HCC.

### 3.5. Nomogram Plot, Drug Sensitivity, and Mutation Analysis

**Figure 5:**
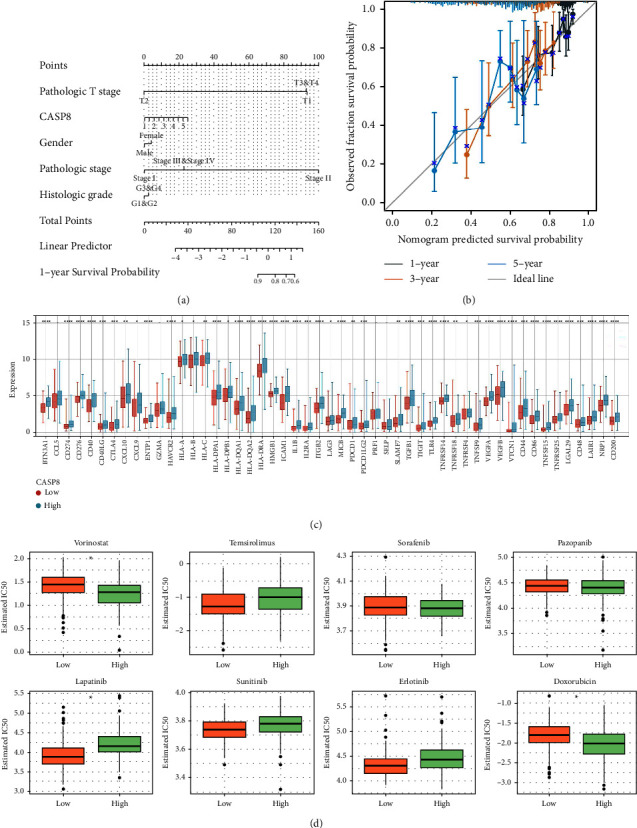
Nomogram plot and drug sensitivity. (a) Nomogram plot of CASP8 combined with clinical features; (b) calibration plots of the constructed nomogram; (c) the expression level of immune checkpoint genes in patients with high and low CASP8 expression; (d) IC50 of specific drugs in patients with high and low CASP8 expression.

**Figure 6:**
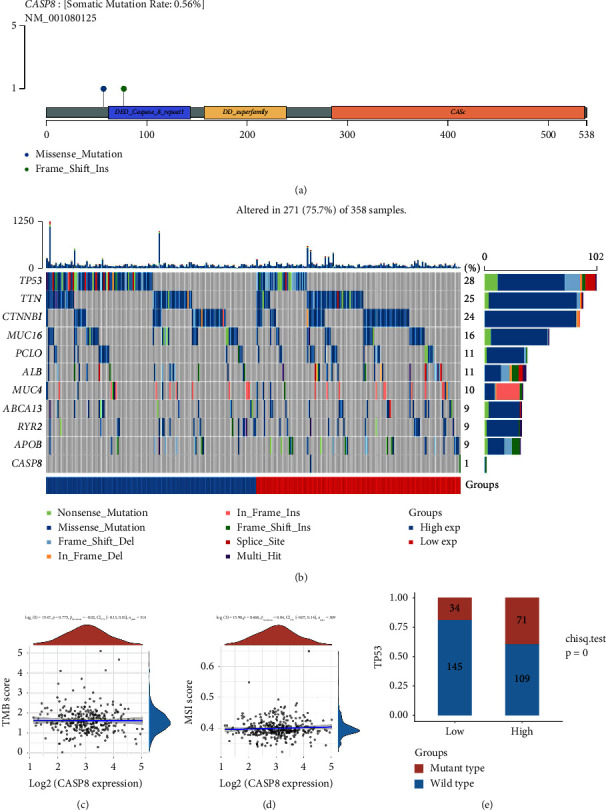
Mutation landscape of CASP8 in HCC. (a) the mutation of CASP8 in the HCC genome; (b) the mutational landscape of HCC; (c–d) correlation between CASP8 and TMB, as well as MSI; (e) patients with high CASP8 expression tend to have more TP53 mutation.

To enhance the clinical prognostic capabilities associated with CASP8, a nomogram plot was developed based on the expression values of CASP8 (Figure 5(a)). Calibration graphs further validated the accuracy of this nomogram, demonstrating a strong correlation between the predicted survival outcomes and the actual observed survival rates (Figure 5(b)). With the increasing relevance of immunotherapy in treating liver cancer, we focused on assessing the impact of CASP8 on the efficacy of such treatments. Our findings revealed that various immune checkpoints exhibited different expression patterns in patients with varying levels of CASP8 expression (Figure 5(c)). This suggests a potential role for CASP8 in modulating the response to immunotherapeutic approaches. Drug sensitivity analysis provided additional insights, indicating that patients with a higher CASP8 expression might exhibit increased sensitivity to certain chemotherapeutic agents, specifically vorinostat and doxorubicin (Figure 5(d)). We also explored the gene mutation landscape of CASP8 in HCC patients (Figures 6(a) and 6(b)). While there was no significant correlation found between CASP8 expression and tumor mutational burden (TMB) or microsatellite instability (MSI) scores (Figures 6(c) and 6(d)), an interesting pattern emerged regarding TP53 mutations. Patients with elevated CASP8 expression tended to have a higher frequency of TP53 mutations (Figure 6(e)). This observation might provide a new perspective on the genetic alterations associated with CASP8 expression in liver cancer.

### 3.6. CASP8 Can Regulate the YEATS2 in HCC

**Figure 7:**
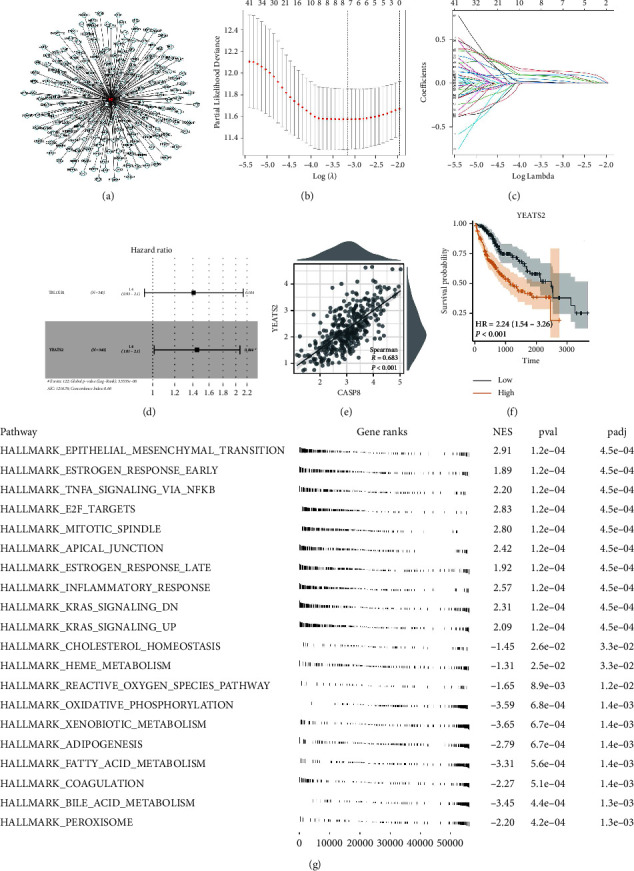
Identification of the CASP8/YEATS2 axis. (a) Top 200 molecules significantly correlated with CASP8; (b–c) LASSO regression analysis based on the molecules significantly correlated with patients survival; (d) multivariate Cox regression analysis of the molecules identified by LASSO regression analysis; (e) correlation between CASP8 and YEATS2; (f) KM survival curves of YEATS2; (g) GSEA of YEATS2 based on the hallmark gene set.

Our analysis has taken initial steps to uncover the potential role of CASP8 in HCC. A key focus was to identify downstream regulatory genes of CASP8. To this end, we first pinpointed the top 200 molecules that showed significant correlation with CASP8 expression (Figure 7(a)). Following this, univariate Cox regression analysis was utilized to determine which of these molecules had a significant correlation with patient survival (Supplementary file 1). Subsequently, we employed LASSO regression analysis to refine our data and optimize the variables for further study (Figures 7(b) and 7(c)). This was an essential step in ensuring the robustness of our findings. The subsequent multivariate Cox regression analysis highlighted YEATS2 as the only molecule significantly and independently correlated with patient survival (Figure 7(d)). Interestingly, a substantial positive correlation between CASP8 and YEATS2 in HCC tissue was observed (Figure 7(e), *R* = 0.683 and *P* < 0.001). This finding indicates a potential interaction or pathway involving these two molecules that could be pivotal in the progression of HCC. Furthermore, our analysis suggested that patients with high CASP8 expression might have a poorer survival outcome compared to those with lower expression levels (Figure 7(f)).

### 3.7. Biological Enrichment and Expression Pattern of CASP8 in HCC

**Figure 8:**
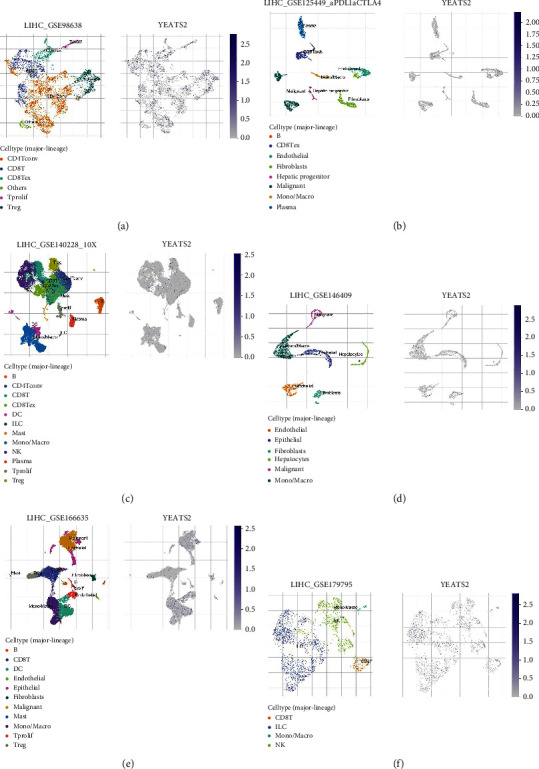
Single-cell analysis of YEATS2 in the HCC microenvironment. (a–f) Expression level of YEATS2 in the HCC microenvironment at the single-cell level.

Delving into the biological role of YEATS2 in HCC, GSEA was conducted. The results indicated that patients with high expression of YEATS2 tended to exhibit enhanced activity in several critical biological processes. Notably, there was an increase in activities related to EMT, early estrogen response, TNF-*α* signaling via the NFKB pathway, E2F targets, and mitotic spindle functions. Conversely, a decrease in activities related to cholesterol homeostasis, heme metabolism, the reactive oxygen species pathway, oxidative phosphorylation, xenobiotic metabolism, and adipogenesis was observed (Figure 7(g)). In addition to the GSEA, single-cell expression analysis was employed to examine the distribution of YEATS2 in HCC. This analysis revealed that YEATS2 is widely distributed across various cell types within HCC, indicating its pervasive influence and potential role in multiple aspects of tumor biology and microenvironment interactions (Figures 8(a)–8(f)). The whole flowchart is shown in Figure S1.

## 4. Discussion

Liver cancer, one of the most common malignant tumors globally, is witnessing an increasing trend in both incidence and mortality rates [33]. This malignancy is mainly classified into primary and secondary types, with HCC being the most prevalent form of primary liver cancer. The development of liver cancer is linked to a variety of factors, including chronic viral hepatitis, alcoholic liver disease, nonalcoholic fatty liver disease, and a range of environmental and genetic contributors [34]. Among the various treatment options, such as surgical resection, radiotherapy, and chemotherapy, targeted therapy emerges as a particularly promising approach [35]. It offers a more precise treatment modality by specifically targeting cancer cells while sparing normal tissues, thereby potentially reducing side effects and improving treatment outcomes. However, the often asymptomatic early stages of liver cancer lead to late diagnoses, challenging the effectiveness of these treatments [36]. Thus, a pivotal focus of future research lies in advancing early detection methods and prevention strategies, alongside enhancing the efficacy and scope of targeted therapies in liver cancer management.

Our study presents a comprehensive analysis of the role of CASP8 in HCC. Initially, we compiled an extensive list of PANoptosis-related molecules and identified CASP8 as a key player through various analyses, including ClueGO and PPI networks. We discovered that high CASP8 expression in HCC patients correlates with poor survival, highlighting its prognostic significance. Furthermore, immunofluorescence images from the HPA database demonstrated CASP8's cellular localization in the nucleoplasm and cytosol, indicating its pivotal role in these regions. Our research extended to a pan-cancer analysis, revealing CASP8's varied expression across different cancer types, and single-cell analysis techniques highlighted its extensive distribution across various cell types. The GSEA provided insights into the biological pathways associated with CASP8, revealing positive correlations with processes such as E2F target activity and inflammatory responses and negative correlations with processes such as myogenesis. In examining the tumor microenvironment, CASP8 showed significant correlations with various immune cell types, suggesting its influence on the immune landscape in HCC. Furthermore, we developed a nomogram plot for better clinical prognostication and investigated CASP8's impact on the efficacy of immunotherapy and chemotherapeutic agents. Mutation analysis revealed a pattern in TP53 mutations among patients with high CASP8 expression. The study also explored the regulatory role of CASP8 on YEATS2 in HCC, finding a strong positive correlation between these molecules. GSEA and single-cell expression analysis of YEATS2 further elucidated its biological role and widespread influence in HCC. Overall, our study provides a detailed insight into the multifaceted role of CASP8 in HCC, emphasizing its potential as a prognostic marker and therapeutic target.

CASP8 is a crucial protein in the human body, playing a pivotal role in the process of apoptosis or programmed cell death [37]. This protein is especially significant as it aids in maintaining the normal life cycle of cells and is vital in eliminating damaged or abnormal cells. In various diseases, the functionality of CASP8 becomes particularly important [38]. For instance, in cancer, aberrant expression or malfunction of CASP8 can lead to tumor cells evading apoptosis, thereby affecting the development and spread of cancer [39]. In addition, CASP8 has shown its significance in certain autoimmune diseases and neurodegenerative disorders, where it may be involved in regulating inflammatory responses or affecting the survival of neuronal cells. Therefore, CASP8 is not only a key element in cellular biology but also a critical target in disease research and potential therapeutic strategies.

YEATS2 is increasingly recognized for its critical role in cellular processes such as gene expression regulation and chromatin structure maintenance [40]. This protein's function is the key in understanding various disease mechanisms, particularly in oncology. For instance, in cancer, dysregulation or mutations of YEATS2 can lead to aberrant gene expression and chromatin modifications, contributing to the oncogenic processes [41]. This involvement in cancer progression underscores the importance of YEATS2 as a potential biomarker for cancer prognosis and a target for therapeutic intervention. Fox example, Zeng et al. indicated that YEATS2 is an underlying biological target for pancreatic cancer and could significantly promote the proliferation and migration ability of cancer cells [42]. Mi et al. found that the YEATS2 is a marker of tumorigenesis for nonsmall cell lung cancer (NSCLC) [43]. Also, YEATS2 is found to be a target for certain drugs. Lan et al. noticed that cinobufacini could delay progression of pancreatic adenocarcinoma by targeting the YEATS2/TAK1/NF-*κ*B axis [44].

An essential aspect of this study involves the data sourced from TCGA patients. It is crucial to acknowledge that a significant proportion of these patients are of Caucasian descent. This demographic skew has potential implications for our findings, as the results may not be wholly representative of diverse populations. Therefore, the conclusions drawn must be cautiously generalized, keeping in mind the racial homogeneity of our primary data source. In addition, the utilization of bioinformatics algorithms in our study warrants careful consideration. While these computational tools are useful in deciphering complex biological data, they are not infallible in capturing the full spectrum of biological significance. The algorithms employed offer an interpretation based on mathematical and statistical principles, which might not always align perfectly with the underlying biological phenomena. Hence, the insights provided by these bioinformatics analyses should be regarded as indicative rather than definitive. They serve as a valuable reference point, guiding further empirical research rather than conclusively delineating biological realities. In summary, while our study offers significant insights, the potential racial bias due to the predominance of Caucasian data in TCGA and the inherent limitations of bioinformatics algorithms as interpretative tools must be considered when applying our findings to broader contexts.

## Data Availability

The data used to support the findings of this study are included within the article/supplementary material, and further inquiries can be directed to the corresponding author.

## Supplementary Materials

Supplementary Materials Figure S1 presents the comprehensive flowchart of our study. Meanwhile, Supplementary File 1 details the univariate Cox regression analysis conducted on CASP8-related molecules, highlighting those that significantly correlate with patient's survival.
