# Supplementary material for: Multidimensional Analysis of PANoptosis-Related Molecule CASP8: Prognostic Significance, Immune Microenvironment Effect, and Therapeutic Implications in Hepatocellular Carcinoma
Source: Genet Res (Camb). 2023 Dec 30;2023:2406193. doi: 10.1155/2023/2406193 (PMC10771335; doi:10.1155/2023/2406193)
Supplement: Supplementary Materials — Figure S1 presents the comprehensive flowchart of our study. Meanwhile, Supplementary File 1 details the univariate Cox regression analysis conducted on CASP8-related molecules, highlighting those that significantly correlate with patient's survival. [file 2406193.f1.pdf]

Figure S1

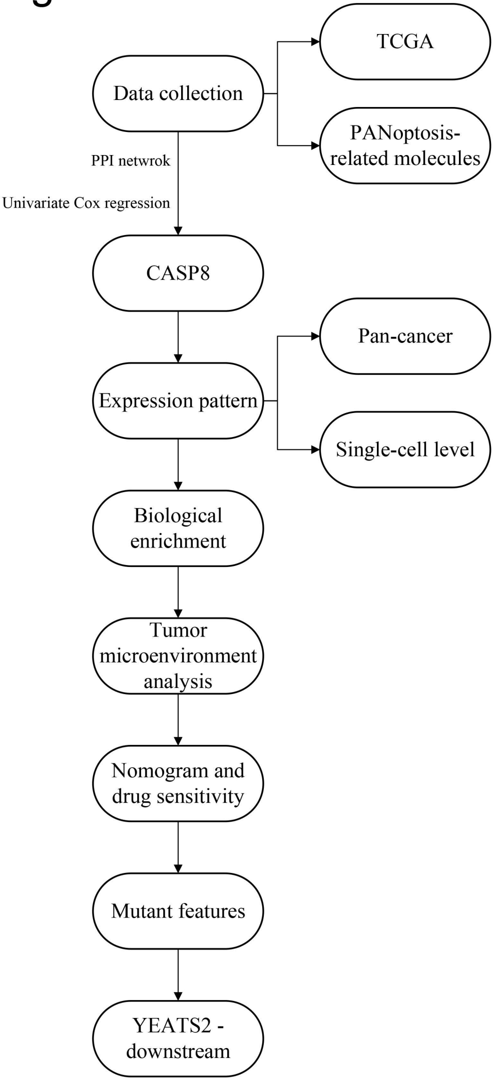

The flowchart of the whole study

Supplementary file 1. The univariate Cox regression analysis of the CASP8-related molecules

id	HR	HR.95L	HR.95H	pvalue
PHF12	1.321458	0.991126	1.761885	0.057534
SCAF11	1.355121	1.05439	1.741625	0.017613
THRAP3	1.418054	1.058571	1.899615	0.019204
MBNL1	1.253631	0.975873	1.610448	0.076919
HNRNPR	1.66335	1.286997	2.149757	0.000101
TBL1XR1	1.910065	1.445046	2.524728	5.46E-06
METAP1	1.440837	1.117538	1.857664	0.004845
GEMIN5	1.679191	1.264444	2.229978	0.000342
MAML1	1.37219	1.081963	1.740267	0.009062
RASA1	1.159432	0.946507	1.420255	0.153027
DR1	1.820679	1.421766	2.331518	2.05E-06
ZSCAN30	1.457455	1.088619	1.951258	0.011396
SLMAP	1.353626	1.020667	1.795201	0.035555
DMTF1	1.224337	0.969661	1.545901	0.088936
FBXO11	1.204793	0.978237	1.48382	0.079613
POLR2B	1.222191	1.00556	1.485491	0.043835
RBM12	1.587988	1.218751	2.06909	0.000615
ZSCAN32	1.330469	0.947687	1.867863	0.099035
MAP4K3	1.30278	1.069518	1.586916	0.008598
QRICH1	1.550003	1.127399	2.131018	0.006971
DUSP11	1.824385	1.336525	2.490323	0.000152
NUP98	1.44916	1.09139	1.924212	0.010333
DDX6	1.298351	0.976872	1.725625	0.072058
NEMP1	1.414475	1.175162	1.702522	0.000246
YEATS2	1.808243	1.422191	2.299089	1.34E-06
BPTF	1.311585	1.048656	1.640437	0.017493
RSRC1	1.30975	1.042086	1.646165	0.020701
PIK3CA	1.44318	1.046149	1.990892	0.02543
SAP130	1.530471	1.177055	1.990002	0.001489
PRMT3	1.705663	1.316367	2.210087	5.36E-05
REV1	1.210014	0.939247	1.558838	0.140211
PHC3	1.200747	0.920227	1.566781	0.177793
KIDINS220	1.283952	1.018288	1.618926	0.034585
HAUS2	1.544902	1.170091	2.039775	0.002156
SLC35F5	1.217535	0.942236	1.57327	0.13232
HNRNPK	1.755325	1.249218	2.466474	0.001186
SRSF1	1.566425	1.147962	2.137429	0.004653
COPB2	1.639603	1.222682	2.198689	0.000957
NIPA2	1.207542	0.967517	1.507113	0.095335
RBBP4	1.373178	1.123828	1.677852	0.001924
XPO1	1.77564	1.321843	2.385227	0.000137
CLASP1	1.221689	0.9269	1.610232	0.155263
REST	1.450128	1.105188	1.902729	0.007327
HPS3	1.204767	1.019522	1.423672	0.028747
ZC3H7A	1.236265	0.948614	1.61114	0.116515
ANAPC1	1.906623	1.363442	2.666203	0.000162
SUPT7L	1.92309	1.401711	2.6384	5.06E-05
UVRAG	1.37094	0.997062	1.885014	0.052155
NPAT	1.363855	1.043635	1.78233	0.02304
TUT4	1.315981	1.061683	1.63119	0.0122
CRLF3	1.398878	1.066777	1.834367	0.015206
CAPRIN1	1.652529	1.274827	2.142135	0.000148
NIPBL	1.4164	1.095437	1.831407	0.007926
LASP1	1.259579	1.031815	1.53762	0.023346
C5orf51	1.548444	1.210176	1.981265	0.000507
HMGXB4	1.492145	1.171375	1.900754	0.001192

TANK	1.466834	1.118374	1.923865	0.005633
ZFC3H1	1.48086	1.165513	1.88153	0.001311
ZFR	1.422747	1.110036	1.823553	0.005364
CCDC93	1.583353	1.236232	2.027941	0.000273
CFLAR	1.107341	0.81175	1.510568	0.51986
RFX5	1.189109	0.973689	1.452188	0.089414
PHF21A	1.670476	1.315723	2.120879	2.52E-05
SMC4	1.312809	1.13594	1.517218	0.000228
SWAP70	1.351129	1.092589	1.670847	0.005484
PIAS3	1.306159	1.073746	1.588878	0.007547
MAPK1	1.337099	1.063722	1.680735	0.0128
NUP107	1.704551	1.332689	2.180174	2.16E-05
ACAP2	1.377994	1.063261	1.785889	0.015366
ZMYM4	1.775362	1.389744	2.26798	4.35E-06
ENOPH1	1.621558	1.25226	2.099762	0.000246
ERBIN	1.145295	0.910783	1.44019	0.245831
UBA3	1.609711	1.173165	2.208699	0.003184
TAF1B	1.839302	1.412081	2.395777	6.22E-06
UTP20	1.516161	1.169431	1.965694	0.001682
ETAA1	1.329021	0.98804	1.787678	0.060051
FOXJ3	1.724097	1.325775	2.242093	4.83E-05
ZBTB11	1.538119	1.15327	2.051394	0.003383
NRAS	1.719563	1.356103	2.180438	7.67E-06
RNF4	1.431793	1.100658	1.862551	0.007481
RAB3GAP1	1.253676	0.937925	1.675723	0.126738
ZNF638	1.446081	1.027578	2.035028	0.034342
DYNC1I2	1.331091	1.079575	1.641204	0.007439
INPP4A	1.471647	1.130854	1.915141	0.004041
CUL3	1.204463	0.956487	1.516728	0.113715
CAB39	1.353529	1.024862	1.787599	0.032925
ZMYM1	1.777509	1.362728	2.318538	2.21E-05
INCENP	1.427561	1.186883	1.717043	0.000158
SMARCC2	1.14669	0.957703	1.372971	0.136321
NAB1	1.248158	1.023639	1.521922	0.02846
SP1	1.338324	1.028194	1.741998	0.030259
JRKL	1.374389	1.098984	1.718811	0.005316
GCC2	1.297172	1.033453	1.628187	0.02485
IWS1	1.343908	0.992968	1.818878	0.055586
USP33	1.455078	1.123168	1.885072	0.004522
PAK2	1.574273	1.194189	2.075328	0.001288
CPSF6	1.89111	1.443538	2.477453	3.76E-06
DCAF17	1.41302	1.065174	1.874459	0.016491
C2CD3	1.454786	1.079191	1.961102	0.013889
CREB1	1.763101	1.311433	2.370327	0.000173
TWF1	1.425007	1.120067	1.812968	0.00394
PRPF38B	1.459478	1.152193	1.848716	0.001722
VPS8	1.597534	1.148907	2.22134	0.005348
PCNP	1.659667	1.254972	2.194866	0.000382
KANSL1	1.513257	1.149626	1.991905	0.003133
KDM3B	1.290328	1.019686	1.632802	0.033814
INO80D	1.506007	1.098094	2.06545	0.011067
RASA2	1.314579	1.016172	1.700617	0.037335
NOP58	1.606443	1.242465	2.077048	0.000299
SNRNP200	1.398207	1.103984	1.770844	0.005426
SLC4A1AP	1.943652	1.361574	2.77457	0.000253
DBR1	1.477173	1.139057	1.915656	0.003264
SLTM	1.179107	0.867923	1.601863	0.291939
GPATCH8	1.227946	0.987697	1.526633	0.064527

ARMC8	1.672852	1.217125	2.299217	0.00152
TOPBP1	1.528746	1.234042	1.893828	0.000102
SP3	1.408373	1.105422	1.794349	0.005588
KPNB1	1.593686	1.254963	2.023832	0.000132
PPHLN1	2.096555	1.490984	2.948082	2.07E-05
RNF214	1.497323	1.12362	1.995316	0.005859
NAA40	1.662079	1.327572	2.080872	9.37E-06
ATP2C1	1.57209	1.224102	2.019005	0.000394
TRIP12	1.478021	1.119301	1.951704	0.005877
RELCH	1.498712	1.11813	2.008832	0.006789
ZCCHC4	1.591115	1.132655	2.235144	0.0074
RNF44	1.369224	1.108438	1.691366	0.003557
ZNF207	1.93745	1.424408	2.635279	2.51E-05
SETD5	1.376363	1.076209	1.76023	0.010924
CNNM4	1.336739	1.071927	1.666973	0.009978
USP24	1.696218	1.286661	2.236141	0.000179
PNPT1	1.704096	1.268143	2.289918	0.000407
CCNT2	1.260518	0.973961	1.631385	0.0785
NR2C2	1.191069	0.956912	1.482525	0.117442
HEATR5B	1.200583	0.931082	1.54809	0.158711
MTREX	1.258148	0.973916	1.625331	0.078804
CDC42SE2	1.398841	1.100092	1.778721	0.006178
RAB10	1.680755	1.335402	2.11542	9.66E-06
TCERG1	1.533004	1.188536	1.977308	0.001002
NEPRO	1.637972	1.19756	2.24035	0.002013
UBE2E1	1.350169	1.097199	1.661463	0.004564
AGFG1	1.785495	1.383448	2.304382	8.45E-06
PPP1R12A	1.309934	1.028338	1.668643	0.028798
SMPD4	1.457878	1.12867	1.883109	0.003891
ADAM10	1.180526	0.934761	1.490906	0.16347
C2orf49	1.571211	1.136617	2.171974	0.006236
ACTL6A	1.44404	1.174301	1.77574	0.000496
UBXN4	1.123558	0.8975	1.406556	0.309418
AGPS	1.673338	1.298532	2.156328	6.92E-05
SRSF10	1.959692	1.426079	2.692973	3.35E-05
PAPOLG	1.674832	1.195948	2.34547	0.002688
ERCC3	1.657044	1.229611	2.233058	0.000907
TMEM131	1.332892	1.038695	1.710415	0.023925
DHX15	1.451631	1.13028	1.864346	0.003509
EIF4G2	1.399808	1.107637	1.769048	0.004865
BTBD10	1.617897	1.250101	2.093904	0.000256
TAOK1	1.359414	1.059567	1.744116	0.015733
UEVLD	1.468141	1.112337	1.937757	0.006692
ZNF621	1.264615	0.986078	1.62183	0.064383
PDCD6IP	1.509095	1.124908	2.024492	0.006049
NEDD1	1.540867	1.230055	1.930217	0.000169
STAMBP	1.465152	1.067269	2.011366	0.018144
PRPF40A	1.510554	1.156912	1.972298	0.002437
STRN	1.516446	1.142055	2.013572	0.004
STK4	1.566185	1.204712	2.036117	0.000805
ATF2	1.434987	1.110231	1.854737	0.005803
TP53BP1	1.332199	1.055061	1.682135	0.015936
DENND5A	1.584682	1.23801	2.02843	0.000257
NPC1	1.55342	1.253172	1.925605	5.84E-05
TTF2	1.584466	1.257284	1.996791	9.62E-05
TRIO	1.37871	1.108317	1.715071	0.003935
CKAP5	1.674361	1.321572	2.121327	1.96E-05
CWC22	1.476262	1.126592	1.934462	0.00474

STXBP3	1.463649	1.151733	1.86004	0.001838
U2SURP	1.440999	1.131964	1.834403	0.003013
ITSN2	1.309942	1.010808	1.6976	0.041227
FAF2	1.630447	1.178346	2.256006	0.003173
ADAM17	1.553654	1.225145	1.97025	0.000278
QTRT2	1.585549	1.181533	2.127715	0.002129
ACTR3	1.581835	1.221007	2.049293	0.000518
ATM	1.22574	0.934162	1.608328	0.141947
RBM12B	1.524999	1.160875	2.003337	0.002432
ATXN2L	1.311782	1.05826	1.626038	0.013259
DDX18	1.531054	1.152491	2.033966	0.003289
COPB1	1.575361	1.213276	2.045505	0.000648
PRKRA	1.468491	1.073154	2.009465	0.016343
VRK2	1.511245	1.207443	1.891487	0.000311
MFN1	1.68932	1.29141	2.209834	0.00013
TOP1	1.471258	1.12405	1.925716	0.004932
TBK1	1.606326	1.114023	2.316186	0.011141
ZC3H15	1.722244	1.268653	2.33801	0.000491
DNAJC13	1.552433	1.203133	2.003143	0.00072
GTF3C3	1.830633	1.337995	2.504656	0.000157
RPE	1.701545	1.285373	2.252465	0.000204
MOB1A	1.435386	1.132201	1.81976	0.00283
NCKAP1	1.407316	1.120066	1.768234	0.003353
ACTR2	1.384545	1.097623	1.746469	0.006031
BAZ2A	1.32742	1.079987	1.631541	0.007122
CAPZA1	1.720945	1.323772	2.237283	5.01E-05
SF3B1	1.395618	1.083017	1.798448	0.009985
FUBP1	1.653749	1.321767	2.069113	1.08E-05
